# Highly efficient *Agrobacterium rhizogenes*‐mediated gene editing system in *Salvia miltiorrhiza* inbred line bh2‐7

**DOI:** 10.1111/pbi.70029

**Published:** 2025-03-26

**Authors:** Mei Tian, Linglong Luo, Baolong Jin, Jianing Liu, Tong Chen, Jinfu Tang, Ye Shen, Haiyan Zhang, Juan Guo, Huawei Zhang, Guanghong Cui, Luqi Huang

**Affiliations:** ^1^ State Key Laboratory for Quality Ensurance and Sustainable Use of Dao‐di Herbs, National Resource Center for Chinese Materia Medica China Academy of Chinese Medical Sciences Beijng China; ^2^ National Key Laboratory of Wheat Improvement Peking University Institute of Advanced Agricultural Sciences, Shandong Laboratory of Advanced Agriculture Sciences in Weifang Weifang Shandong China

**Keywords:** *Salvia miltiorrhiza*, CRISPR/Cas9, genomic editing system, *Agrobacterium rhizogenes*

## Abstract

The CRISPR/Cas9 system is a powerful tool for genomic editing with significant potential for gene function validation and molecular breeding in medicinal plants. *Salvia miltiorrhiza*, a model medicinal plant, was among the pioneers to utilize CRISPR/Cas9 technology, though achieving high‐efficiency homozygous knockout mutants has been challenging. In this study, the analysis of variations at 241 single‐guide RNA (sgRNA) across different reference genomes and experimental materials was conducted first, leading to the identification of the six‐generation inbred line bh2‐7 as the most suitable reference genome and experimental material for gene editing research in *S. miltiorrhiza*. Next, five *Agrobacterium rhizogenes* strains were evaluated for hairy root induction, editing efficiency, and mutation patterns, with C58C1 and K599 emerging as the most effective delivery systems. Using the CRISPR/Cas9 vector pZKD672, 53 target sites were successfully edited, with K599 achieving 71.07% editing efficiency and 36.74% homozygous or biallelic (HOM) efficiency and C58C1 showing 62.27% editing efficiency and 23.61% HOM efficiency. We thus constructed a large‐scale mutant library targeting 121 genes with 170 sgRNAs, yielding 1664 homozygous or biallelic mutants. Analysis of 65 low‐efficiency target sites revealed that sgRNA mismatches and secondary structures were key factors reducing HOM efficiency, offering insights for future target design. This study establishes an efficient CRISPR/Cas9 system, advancing precision breeding and metabolic engineering research in medicinal plants.

## Introduction

Medicinal plants are a valuable source of compounds with significant importance in medicine, economics, ecology, and botany. However, significant bottlenecks persist in the exploration and utilization of key genes responsible for the biosynthesis of bioactive compounds (Hu *et al*., 2021; Sun *et al*., 2022). The CRISPR (clustered regularly interspaced short palindromic repeat)/Cas9 (CRISPR‐associated protein 9) system, a powerful genome editing tool (Jinek *et al*., 2012), has demonstrated considerable potential in enhancing the production of medicinal ingredients, validating gene function, and facilitating targeted modification of traits in medicinal plants. Nevertheless, editing efficiency remains a limited factor in many medicinal plant species (Alagoz *et al*., 2016; Kui *et al*., 2017; Li *et al*., 2017, 2021; Shi *et al*., 2020). This limitation can be partially attributed to the underdeveloped molecular biology research foundation for many of these plants, including the lack of mature and effective genetic transformation systems, homozygous inbred varieties, and large‐scale studies on gene mutation efficiency in specific species.

*Salvia miltiorrhiza*, a model system for medicinal plant biology, was one of the earliest medicinal species subjected to CRISPR/Cas9 genomic editing. This technology has been employed to elucidate biosynthesis pathways and regulatory factors governing active compounds, notably tanshinones and salvianolic acids (Li *et al*., 2017; Shao *et al*., 2024; Zhou *et al*., 2018). Five genome sequences have been published (Ma *et al*., 2021; Pan *et al*., 2023; Song *et al*., 2020; Xu *et al*., 2016; Zhang *et al*., 2015), each using different varieties. Consequently, a standard variety for scientific research has yet to be established. While the highest reported mutant efficiency in *S. miltiorrhiza* reaches 90% (Li *et al*., 2017; Tang *et al*., 2019), the homozygous mutation efficiency remains relatively low, with a maximum reported efficiency of 50% (Zhou *et al*., 2018). In plants, *Agrobacterium*‐mediated delivery of CRISPR/Cas9 components is a widely used method for rapid, precise, cost‐effective, and relatively straightforward ‘in root’ functional analysis of target genes (Cao *et al*., 2022, 2024; Fan *et al*., 2020; Niazian *et al*., 2022; Zhang *et al*., 2023a; Zhou *et al*., 2018). However, only a limited number of *A. rhizogenes* strains have been reported for use in *S. miltiorrhiza*, including C58C1, ATCC15834, ACCC10060, and A4. While these strains have been primarily used to analyze hairy root induction rates, their comparative editing efficiencies have been less thoroughly investigated. Therefore, for medicinal plants, selecting a widely accepted reference genome and stable experimental varieties, together with identifying effective CRISPR/Cas9 delivery systems are crucial for further large‐scale genome engineering research.

In the CRISPR/Cas9 system, the first 20 nucleotides at the 5′ end of the sgRNA make up the variable spacer sequence, which provides sequence specificity to the endonuclease Cas9 by the virtue of its complementarity to the target sequence. An important feature of the SpCas9 system is the protospacer adjacent motif (PAM) site (NGG for SpCas9) (Mojica *et al*., 2009). While the invariable, about 80‐nt long 3′section of the sgRNA (Ma *et al*., 2015), called the scaffold sequence, consists of four structural units: the repeat–anti‐repeat duplex (RAR) and stem loop 1–3 (SL1, SL2 and SL3), mediating the interactions with the SpCas9 protein (Liang *et al*., 2016; Ma *et al*., 2015; Sagarbarria *et al*., 2023). They determined the editing efficiency of CRISPR/Cas9 in plants. However, choosing an optimum sgRNA sequence has its challenges. Mismatches in the PAM site or between the sgRNA and the target DNA can lead to decreased or failed mutagenesis efficiency (Liu *et al*., 2015). Another common consequence of these mismatches is the off‐target effect. Off‐target sites often have only a few mismatches compared to the target sequence and are frequently located in protein‐coding regions, often in homologues of target gene (Bravo *et al*., 2022; Sturme *et al*., 2022). This is particularly relevant as genes involved in the biosynthesis pathways of active ingredients often exhibit high sequence similarity. In addition to the aforementioned factors that affect the efficiency of CRISPR/Cas9, numerous studies have screened large gRNA libraries and developed algorithms to predict gRNA sequence‐dependent activity. But currently, most research is based on animal models (Graf *et al*., 2019; Huszár *et al*., 2023; Konstantakos *et al*., 2022; Tálas *et al*., 2021; Wong *et al*., 2015). Research on large‐scale target testing and sequence preferences in plants remains limited (Ma *et al*., 2015; Naim *et al*., 2020). Therefore, comprehensive large‐scale target testing in plants is critical to identify plant‐specific factors affecting CRISPR/Cas9 editing efficiency, and facilitate the development of optimized gRNA design strategies adapted to botanical systems.

**Figure 1:**
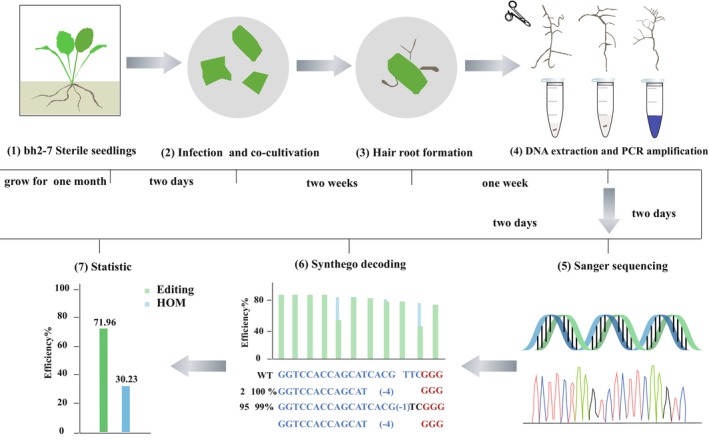
An efficient *A. rhizogene*‐mediated genetic transformation protocol in *S. miltiorrhiza* including seven steps: (1) bh2‐7 sterile seeding; (2) Infection and co‐cultivation; (3) Hair root formation; (4) DNA extraction and PCR amplification; (5) Sanger sequencing; (6) Synthego decoding; (7) Statistic.

In this study, we designed 241 sgRNAs targeting 131 genes based on the genome sequence of the *S. miltiorrhiza* inbred line bh2‐7 (Chen *et al*., 2024). This line, which has undergone six generations of self‐pollination, exhibits the lowest heterozygosity rate among five sequenced varieties (Ma *et al*., 2021). By analysing sequence variations within these sgRNA target sites in both a purple‐flowered variety (ZH wild type) and bh2‐7, we confirmed that bh2‐7 is an ideal material for subsequent research. We then systematically evaluated the hairy root induction capability, editing efficiency, and mutation patterns of five *A. rhizogenes* strains (C58C1, K599, Ar.Qual, MSU440, and Ar1193) using multiple sgRNA targets and generating nearly 2000 hairy roots. Upon this high‐efficiency system, we further analysed the editing efficiency of 170 target sites in *S. miltiorrhiza* and identified several factors contributing to low CRISPR/Cas9 activity. These findings provide critical insights into the sequence and structural determinants of CRISPR/Cas9 efficiency in *S. miltiorrhiza* and offer guidance for optimizing gRNA design in plant genome editing (Figure 1). As a result, we achieved a remarkable 100% target editing efficiency and 70% HOM efficiency, currently representing the highest reported editing efficiency in this species.

## Results

### Natural variation in two *S. miltiorrhiza* germplasm: Purple‐flowered and bh2‐7

**Figure 2:**
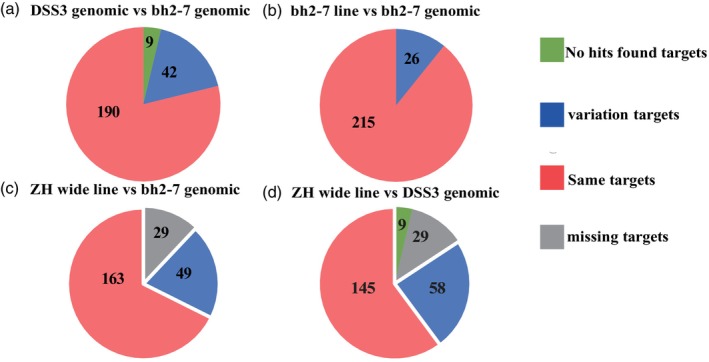
Variation of 241 guide sequences across different materials and genomes. (a) Variation of 241 guide sequences in DSS3 and bh2‐7 genomic. (b) Variation of 241 guide sequences in bh2‐7 line and bh2‐7 genomic. (c) Variation of 241 guide sequences in ZH wide type and bh2‐7 genomic. (d) Variation of 241 guide sequences in ZH wide type and DSS3 genomic.

The heterozygosity of the four previously reported *S. miltiorrhiza* genomes is 2–4 times higher than that of the inbred line bh2‐7 (Ma *et al*., 2021; Pan *et al*., 2023; Song *et al*., 2020; Xu *et al*., 2016; Zhang *et al*., 2015). Therefore, to minimize the potential impact of plant heterozygosity on editing efficiency, we designed 241 sgRNAs targeting 131 genes based on the bh2‐7 genome to assess the genetic variation between *S. miltiorrhiza* germplasms (Table S1). We first compared the variation of 241 target sites between the DSS3 and bh2‐7 genome, revealed that 190 target sites were identical between the two genomes, while 42 target sites exhibited 1–4 SNPs. Notably, 9 target sites could not be mapped to the DSS3 genome (Figure 2a, Table S2). Subsequently, we performed PCR amplification of the 241 target sites using both the bh2‐7 line and ZH wild type as materials to validate the genomic sequences. Analysis of the 241 sgRNA target sites in cultured bh2‐7 seedlings showed a much higher degree of sequence conservation: 215 targets (89.21%) matched the reference genome, with only 26 targets showing variations (10.79%) (Figure 2b; Table S2). In contrast, sequencing PCR products from the ZH wild variety revealed significant genetic variations compared to bh2‐7 genomic (Figure 2c; Table S2). Notably, 29 target sequences (12.03%) could not be amplified from the ZH wild variety using primers designed based on the bh2‐7 genomic sequence (Table S2). An additional 49 (20.33%) target sequences exhibited sequence variations, and only 163 targets (67.63%) matched the bh2‐7 genome.

We further compared the genetic variations of 241 target sites between the ZH wild type and DSS3 genomes. Excluding the 29 targets that could not be amplified from the ZH wild type, 145 targets (60.17%) matched the DSS3 reference genome, while 58 targets (24.07%) exhibited variations (Figure 2d). Notably, the number of variant target sequences was higher when using DSS3 as reference genome than bh2‐7 genome. Additionally, 9 target sites that could not be mapped to the DSS3 genome were successfully amplified from the ZH wild type, with the majority (7/9) matching the bh2‐7 genome (Table S2). These analysis revealed that bh2‐7 possesses high genetic stability and uniformity, making it an ideal candidate for CRISPR/Cas9‐based genomic editing studies.

### Effects of different *A. rhizogenes* strains on the CRISPR/Cas9 editing efficiency in bh2‐7

**Figure 3:**
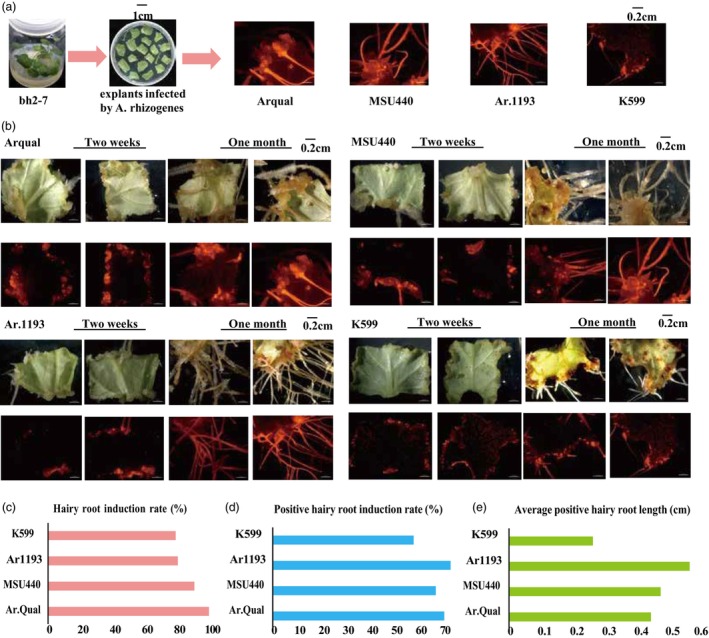
The hairy roots induced by four different *A. rhizogenes* strains in bh2‐7. (a) Schematic representation of hairy root induction in bh2‐7 using four different *A. rhizogenes* strains. (b) Hairy roots induced by various *A. rhizogenes* strains in bh2‐7, with transgenic roots emitting red fluorescence at 558 nm due to the expression of DsRed protein. (c–e) Effects of different *A. rhizogenes* strains on hairy root induction rate (c), positive rate (d) and the length of positive hairy root (e) in bh2‐7 explants.

We initially compared the performance of different *A. rhizogenes* strains (Ar.Qual, MSU440, Ar1193 and K599) in inducing hairy roots in bh2‐7 explants using the pW501 vector containing the DsRed reporter gene (Pan *et al*., 2022b). Leaf explants from healthy bh2‐7 seedlings were infected with each strain and cultured in the dark at 25 °C. Within 2 weeks, hairy roots emerged from explants infected with all strains except K599. After 1 month, significant root elongation was observed in the four strains that produced roots (Figure 3a). Under handheld fluorescence and confocal microscope, explants exhibiting red fluorescent signals, indicative of successful transformation (positive hairy roots), were distinguished from those lacking red fluorescence (Figure 3b). All four strains  exhibited robust hairy root induction capacity (Figure 3c,d), with over 79% of explants (30 per strain) generating positive hairy roots, and the positive hairy root induction rate exceeding 58% (Figure 3d). The transgenic roots averaged 0.4 cm in length, whereas auxin‐deficient strain K599 exhibited reduced growth (0.26 cm) (Figure 3e).

**Figure 4:**
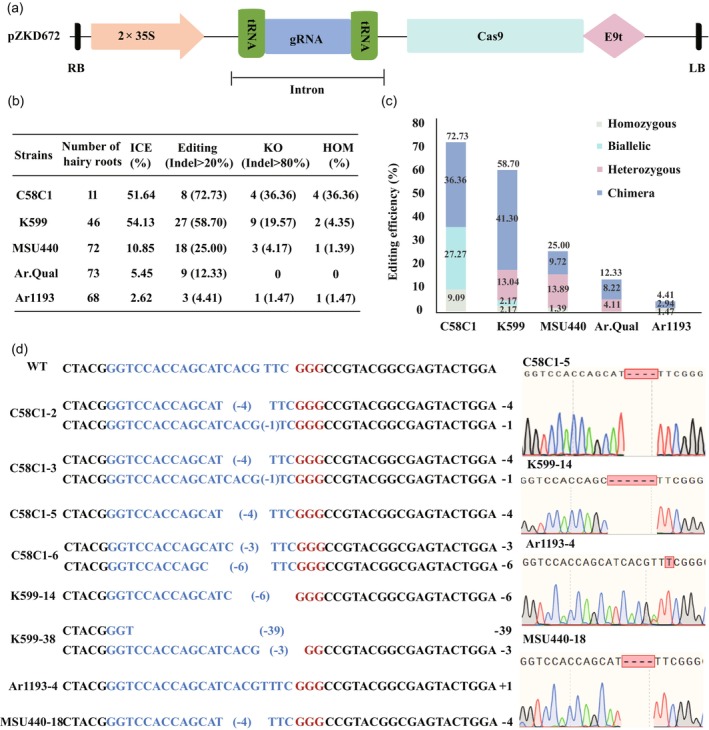
Diagram of pZKD672 for CRISPR/Cas9 in bh2‐7 and the mutant types verified by sequencing. (a) Schematic representation of partial pZKD672 plasmid, including Cas9 and gRNA expression cassettes (Pan *et al*., 2022a). (b) ICE, editing efficiencies, KO efficiencies, HOM efficiencies of five *A. rhizogenes* strains. (c) Editing efficiencies of different mutation types generated by five *A. rhizogenes* strains. (d) Homozygous or biallelic mutants generated by different *A. rhizogenes*. The PAM sequence “GGG” is highlighted in red, and the target sequence is shown in blue. The sequencing peaks corresponding to homozygous mutants are displayed on the right side of the sequence.

To evaluate Cas9‐sgRNA‐mediated precise genome editing using the above *A. rhizogenes* strains and the previously reported C58C1 (Zhou *et al*., 2018), the pZKD672 vector was employed. This vector uses an intron‐mediated 35S enhancer strategy to express both Cas9 and sgRNA from a single expression cassette (Figure 4a) and has been previously shown to achieve a high editing efficiency of 81% in lettuce (Pan *et al*., 2022a). A 20‐nucleotide guide sequence targeting the *SMILTO09119* gene was constructed into the pZKD672 vector and transformed into the five *A. rhizogenes* strains, using bh2‐7 as the infection material. PCR products covering the predicted target site, with a fragment size of approximately 500 bp, were sequenced and genotyped using the Synthego ICE analysis tool (Develtere *et al*., 2024). Based on the genotype and indel scores provided by Synthego for each mutant, six parameters were evaluated for the five *A. rhizogenes* strains (Figure 4).

C58C1 and K599 demonstrated superior ICE efficiencies, both exceeding 50%. In contrast, MSU440, Ar.Qual and Ar1193 exhibited significantly lower ICE efficiencies (2%–10%), suggesting a smaller proportion of cells with indels. C58C1 exhibited the highest editing, KO, and HOM efficiencies, achieving 72.73% editing efficiency (8 out of 11 hairy roots) and 36.36% for both KO and HOM efficiency (Figure 4b,c). Notably, all four mutants with indel >80% in C58C1 were either homozygous (C58C1–5) or biallelic (C58C1–2, 3, 6) (Figure 4d). Additionally, chimeric mutants were observed in C58C1 at efficiencies of 36.36% (Figure 4c; Table S3). K599 followed with 58.7% editing efficiency (27/46), encompassing various mutation types: homozygous (1, 2.17%), biallelic (1, 2.17%), heterozygous (6, 13.04%), and chimeric mutations (19, 41.3%) (Figure 4c; Table S3). Nine mutants exhibited indel efficiencies >80%, contributing to a KO efficiency of 19.57%. Two mutants, K599‐14 and K599‐38, had high indel percentages (98% and 83%, respectively), and were identified as HOM mutants (Figure 4d).

Despite analysing more than 60 hairy roots for each of the remaining three strains (MSU440, Ar.Qual and Ar1193), they displayed lower ICE efficiencies (2.62%–10.85%) and correspondingly lower editing efficiencies: 25% (18/72), 12.33% (9/73), and 4.41% (3/68), respectively. Heterozygous and chimeric mutations were detected in all three strains, but only MSU440‐18 and Ar1193‐4 produced homozygous mutants, resulting in low HOM efficiencies of 1.39% (1/72) and 1.47% (1/68), respectively (Figure 4b,d). Mutants with indel efficiencies >80% were scarce in these strains: 3 (4.17%) in MSU440, 1 (1.47%) in Ar1193, and none in Ar.Qual, leading to significantly lower KO efficiencies compared to C58C1 and K599.

Analysis of 136 editing sites generated by the five *A. rhizogenes* strains revealed 28 distinct editing types. Single base deletions (25%) and four base deletions (20.59%) were the most prevalent (Figure S1). The cucumopine‐type strain K599 exhibited the highest diversity in mutation types, while the four agropine‐type *A. rhizogenes* strains, including C58C1, showed similar editing patterns, primarily involving in deletions of one or four bases, and insertions greater than nine bases. In summary, C58C1 and K599 emerged as the most efficient strains for genome editing, showcasing high editing and HOM efficiencies.

### Screening different sgRNAs for multiplex mutagenesis with K599 and C58C1

**Figure 5:**
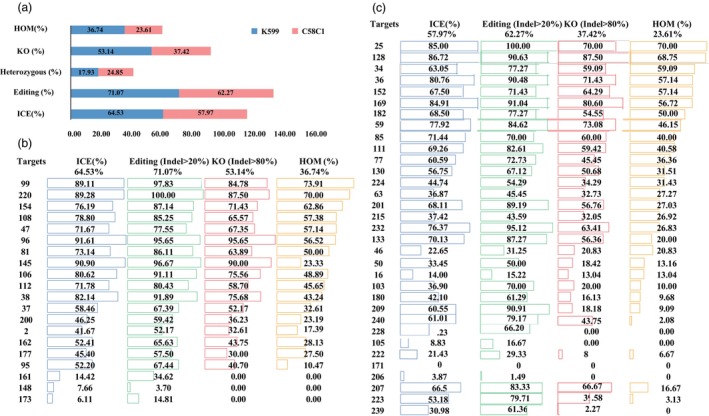
Editing efficiency of 20 targets for K599 and 33 targets for C58C1. (a) Average ICE, editing, KO, and HOM efficiency (homozygous or biallelic mutant) for 20 targets in K599 and 33 targets in C58C1. (b) ICE, editing, KO, and HOM efficiency for each of the 20 targets in K599. (c) ICE, editing, KO, and HOM efficiency for each of the of 33 targets in C58C1.

Although the target gene *SMILTO09119* exhibited higher editing efficiency in both K599 and C58C1, it still did not achieve the desired level of efficiency. Therefore, we expanded the target screening to include 53 sgRNAs for a more comprehensive evaluation. In K599, 20 sgRNAs yielded 909 hairy roots, while in C58C1, 33 sgRNAs produced 1296 hairy roots, with an average of 30–40 hairy roots for each target. Both strains exhibited a wide range of ICE efficiencies (approximately 4%–100%), with average efficiencies of 64.53% for K599 and 57.97% for C58C1 (Figure 5; Table S4). The average editing efficiencies were also high, reaching 71.07% for K599 and 62.27% for C58C1. Notably, sgRNA‐220 in K599 and sgRNA‐25 in C58C1 achieved 100% editing efficiency. Among the edited mutants, 483 in K599 and 485 in C58C1 exhibited indel efficiencies >80%, indicating KO efficiencies of 53.14% and 37.42%, respectively. Further analysis of these KO mutants revealed similar average efficiencies for homozygous or biallelic mutants in both strains, approximately 20%–30%, suggesting that over half of the mutants with indel efficiencies >80% were homozygous or biallelic (Figure 5; Table S4). The highest HOM efficiencies were observed with sgRNA‐99 in K599 (73.91%) and sgRNA‐25 in C58C1 (70%).

Although the editing and HOM efficiency of the K599 strain were slightly superior to those of C58C1, its prolonged induction period and increased susceptibility to contamination during hairy root transformation rendered it less suitable for large‐scale applications. Consequently, the C58C1 strain was selected for further knockout 117 sgRNAs to construct a mutant hairy root library. A total of 5944 hairy roots were screened, with an average of 48 independent hairy root lines generated per target, which was relatively higher than the 33 targess testing. Mutations were successfully detected in 105 target sites, resulting in 3143 editing events, and 1024 homozygous or biallelic mutants were obtained, with an average editing and HOM efficiency of 52.88% and 17.23%, respectively, which was relatively lower than the 33 targess testing (Table S5). In total, we successfully obtained 1664 homozygous or biallelic mutants targeting 121 genes with 170 targets, which provide a robust platform for functional genomics studies and metabolic network analysis in *S. miltiorrhiza* (Table S6).

### Factors affecting CRISPR/Cas9 editing efficiency in *S. miltiorrhiza*

**Figure 6:**
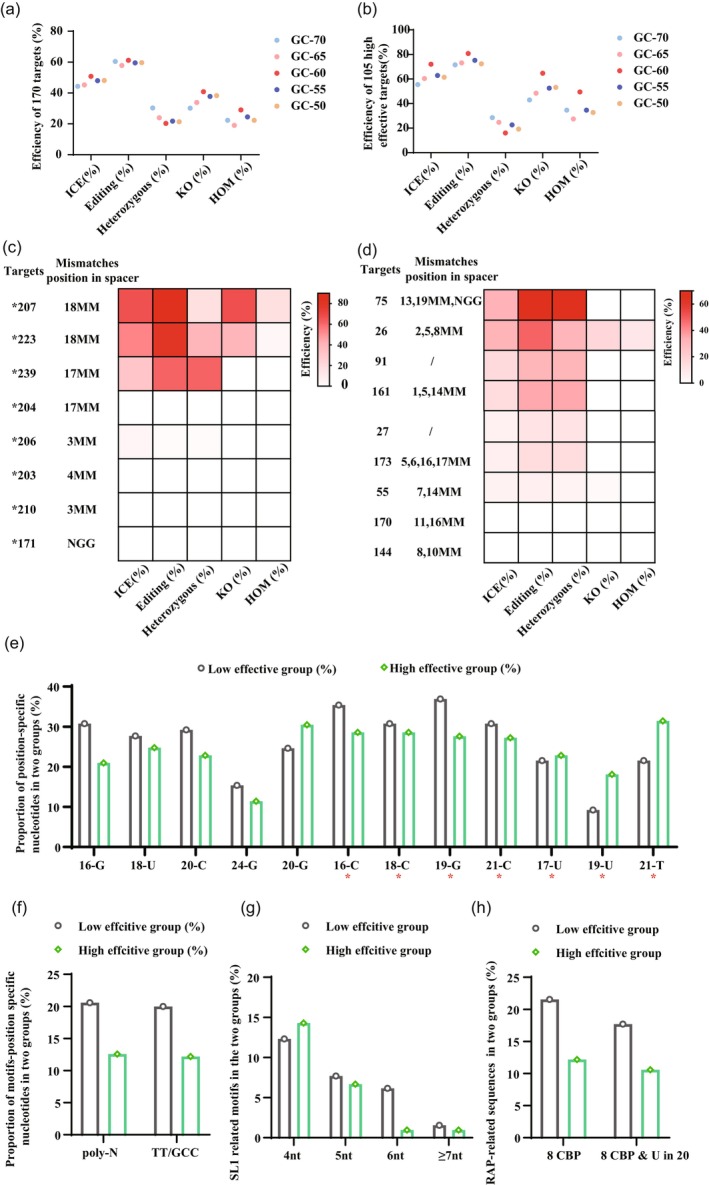
Factors affecting CRISPR/Cas9 editing efficiency in *S. miltiorrhiza*. (a) Efficiency of targets with varying GC contents among 170 targets. (b) Efficiency of targets with varying GC contents among 105 high‐efficiency targets. (c) Analysis of target editing efficiency for eight spacer mutations. (d) Analysis of target editing efficiency for nine spacers with homologous sequence. (e) Proportion of position‐specific nucleotides in two groups, and sequence position ordering: 5′‐20 nt‐NGG‐3′. The red asterisks (*) indicate position‐specific nucleotides in the *S. miltiorrhiza* sgRNA design. (f) Proportion of motifs‐position specific nucleotides in two groups. (g) Frequency of presence of SL1‐related sequences in both two groups (%). (h) Frequency of presence of RAP‐related sequences among two groups (%).

While the CRISPR/Cas9 system demonstrated high overall editing efficiency, significant variability was observed across targets. Among 170 sgRNAs, 65 sgRNAs exhibited HOM efficiency below 10%, which provided critical insights into factors influencing CRISPR/Cas9 performance. In plants, sgRNAs with a GC content greater than 50% are generally reported to exhibit higher effectiveness (Liang *et al*., 2016). Among the 170 sgRNAs analysed in this study, when the GC content of sgRNAs was 60%, the editing, knockout (KO), and HOM efficiencies were significantly higher (Figure 6a). This trend was particularly pronounced in the 105 high‐effecitive target groups (Figure 6b). However, this pattern was not observed in the lower‐effecitive group (Figure S2), indicating that GC content alone may not fully explain the variation in editing efficiency and prompting further investigation into other influencing factors.

Nucleotides adjacent to the PAM sequence, particularly spaning positions 16–20 of the sgRNA, are critically important due to their essential role in target recognition and Cas9 loading (Konstantakos *et al*., 2022; Liu *et al*., 2015; Modrzejewski *et al*., 2020; Wong *et al*., 2015). Among 65 low‐HOM sgRNAs, eight exhibited mismatches in the spacer or PAM region (Table S6). Distal mismatches (17–18MM from PAM) demonstrated higher tolerance than proximal (3–4MM) or PAM (NGG) mismatches (Figure 6c). Additionally, the presence of homologous genes significantly contributes to off‐target effects and reduced editing efficiency in the CRISPR‐Cas9 system (Modrzejewski *et al*., 2020). Nine sgRNAs, with 1–10 homologous genes sharing 84%–97% sequence similarity, resulted in zero HOM efficiency. These homologous genes nearly shared 16–20 identical bases within the 20‐nt guide sequence and possessed the same PAM sequence (Table S6, Figure 6d). Overall, consistent with previous findings, mismatches in the PAM region and proximal nucleotides (3–4MM), as well as the presence of homologous genes, increase the risk of off‐target effects and significantly reduce editing and HOM efficiency.

Single‐guide RNA sequence features, particularly those within the PAM and its adjacent regions16–20 of the gRNA, have been demonstrated to significantly influence CRISPR/Cas9 activity, although these features may vary across different genomic contexts, including human, *E. coli*, mouse, and zebrafish datasets (Graf *et al*., 2019; Moreb and Lynch, 2021). Guanine (G), uracil (U), cytosine (C), and guanine (G) at positions 16, 18, 20, and 24, respectively, are disfavored, while guanine (G) at position 20 is preferred (Chari *et al*., 2015; Doench *et al*., 2014), with consistent trends observed in *S. miltiorrhiza*. However, in *S. miltiorrhiza*, some findings differ from previously reported results (Wang *et al*., 2019; Wong *et al*., 2015). Nucleotides such as C at positions 16, 18, 21, as well as G at 19, are more frequently associated with low‐efficiency groups. In contrast, U at position 17 and 19, thymine (T) at 21 are more prevalent in high‐efficiency groups. Notably, G at position 19 (36.92%), C at 16 (35.38%) and 21 (30.77%) showed the highest frequencies in the low‐efficiency group, while T at 21 (31.43%) exhibited the highest frequency in the high‐efficiency group. These results provide new evidence for designing highly efficient target sites in *S. miltiorrhiza* (Figure 6e). Additionally, repetitive bases, such as poly‐N motif, ‘TT‐motif’ and ‘GCC‐motif’, can also reduce cleavage efficiency (Graf *et al*., 2019). Consistent with previous studies, these motifs were more frequently observed in the low‐efficiency group in *S. miltiorrhiza* (Figure 6f, Table S6).

Recent research reported some motifs that are complementary to the RAR unit and SL1 interfere with sgRNA loading and target binding, respectively (Huszár *et al*., 2023). Consistent with previous studies, these SL1‐complementary spacer motifs are more frequently observed in low‐efficiency target sites, particularly when the complementary sequence length increases to 5–8 nucleotides (Table S6, Figure 6g). Similarly, the occurrence of U at position 20 often pairs with A at position 51, leading to the disruption of the stem‐loop RAP structure (involving more than seven consecutive base pairs) (Figure S3). These phenomena are also more prevalent in low‐efficiency group (Table S6, Figure 6h). In summary, through analysis of 65 low‐effective targets, 58 targets (89.23%) can be attributed to one or five factors. Except for these 58 low‐effective targets, the average editing efficiency can reach 71.96%, with homozygous or biallelic efficiencies at 30.23% (Table S6).

### Analysis of mutation types caused by CRISPR/Cas9 in *S. miltiorrhiza*

**Figure 7:**
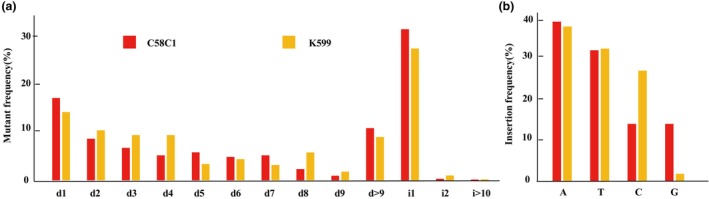
Different mutation types in K599 and C58C1. (a) Mutation types and frequencies of biallelic or homozygous mutants from K599 and C58C1. (b) Frequency of occurrence of four nucleotides in one base insertion type.

To gain a deeper understanding of mutation patterns, the analysis was initially expanded to include 351 biallelic and 289 homozygous mutants derived from 53 sgRNAs in both K599 and C58C1. This larger dataset revealed striking similarities in the mutation patterns of C58C1 and K599, with both strains predominantly exhibiting insertions of one or two nucleotides and deletions of varying lengths (Figure 7a; Table S7). Furthermore, 150 sgRNA induced by C58C1 showed similar mutation patterns (Figure S4). Small deletions (1–9 bp) or single‐base insertions accounted for 71.29% of mutants in K599 and 67.98% in C58C1, aligning with previous plant studies, where CRISPR/Cas9‐induced NHEJ‐midiated mutations typically involve small deletions or single base insertions (Bortesi *et al*., 2016; Elorriaga *et al*., 2018). Deletions longer than 9 bp, even 15–40 bp (10.88% in C58C1 and 9.04% in K599) (Table S7), were also observed. These less frequent longer deletions may represent the outcome of MMEJ, another DSB repair pathway reported in plants, human, and mouse cell lines (Li *et al*., 2015).

A significant proportion of mutation events were single‐base insertions, accounting for 27.51% in K599 and 31.55% in C58C1. Analysis of the base composition of these 1‐bp insertions revealed a predominance of A or T insertions, with A + T insertions comprising approximately 70% (70.91% in K599 and 71.72% in C58C1) (Figure 7b; Table S7). This observation aligns with previous reports in rice (88.2%) (Zhang *et al*., 2014) and Arabidopsis (54.1%) (Ma *et al*., 2015).

## Discussions

*Salvia miltiorrhiza* is a highly valued medicinal herb with widespread use in traditional Chinese medicine and other applications (Fang *et al*., 2018; Wang and Peters, 2022). Here, we demonstrate that the inbred line bh2‐7, having undergone six generations of self‐pollination, is a suitable resource for gene editing. Through a comprehensive analysis of 121 genes (170 targets) and nearly 8149 hairy root samples, we successfully introduced mutations in 112 genes (157 targets), generating a total of 4596 mutant lines. Further detailed analysis revealed that 103 genes (132 targets) produced 1664 homozygous or biallelic mutants, achieving a maximum editing efficiency of up to 100% and a homozygous or biallelic (HOM) efficiency of 70%. By systematically analysing 65 low‐efficiency target sites, we identified several *S. miltiorrhiza*‐specific sgRNA design principles. These findings provide both theoretical insights and practical strategies for optimizing genome editing in *S. miltiorrhiza* and potentially other plant species, advancing the field of precision genetic engineering.

*Salvia miltiorrhiza* presents challenges in obtaining inbred lines due to severe inbreeding depression. Among the five whole genome assemblies available, only two utilized inbred lines, DSS3 from Mount Tai (three generations of self‐pollination) (Song *et al*., 2020), and bh2‐7 (six generations), used in this study, which originated from a planting base in Laiwu, Shandong Province (Ma *et al*., 2021). Heterozygosity analysis, based on 17‐mer depth distribution or Illumina read mapping, consistently indicates that bh2‐7 has lower heterozygosity compared to the other four lines (Ma *et al*., 2021). The variation analysis based on 241 target sites (Table S2) further supports the low heterozygosity of bh2‐7, indicating that its genetic background is more uniform and stable. Although we used bh2‐7 with the lowest heterozygosity, there are still eight target sites that showed variations compared to the target sequence out of the 170 tested sites. Our results align with previous findings, showing a stronger tolerance for PAM‐distal mismatches compared to mismatches within the PAM or PAM‐proximal (1–4MM) region (Table S6). This may be related to the conformation of the guide RNA–DNA duplex and Cas9 activation (Bravo *et al*., 2022). Notably, PAM‐distal mismatches reduced HOM efficiency without affecting editing efficiency, suggesting that ideal sgRNAs should avoid all mismatches (Figure 6d). Thus, for highly heterozygous medicinal plants, obtaining ideal material and reference genome with relatively low heterozygosity through methods such as constructing inbred lines or haploids is vital to ensure efficient gene editing. On the contrary, simultaneously editing a target gene using two or more target sites can significantly increase the likelihood of achieving effective mutations. In the 121 genes we studied, 49 genes were designed with two target sites. The results showed that only one gene failed to produce mutants, meaning both target sites for this gene were unsuccessfully edited. In contrast, among the 72 genes with only a single target site, 7 genes (6%) failed to produce any mutants. These findings highlight the greater reliability and effectiveness of dual‐target designs in achieving successful gene editing outcomes. This strategy has also been successfully applied to highly heterozygous species, such as cotton (*Gossypium spp*.) (Gao *et al*., 2017) and poplar (*Populus spp*.) (Wang *et al*., 2020).

Efficient delivery of CRISPR/Cas9 components into cells is also a crucial factor for successful gene editing (Jedličková *et al*., 2024). We explored the effects of five different *A. rhizogenes* strains on hairy root induction, gene editing efficiency, and mutant types in bh2‐7. All strains showed relatively high positive hairy root induction rates and similar editing types (Figure 3; Figure S1). However, K599 and the commonly used C58C1 emerged as the most efficient strains. Beyond demonstrating high editing efficiency in *S. miltiorrhiza* (Figure 5), K599 was also widely used in cut‐dip‐budding gene delivery systems without tissue culture or other gene editing systems, especially for species with challenging genetic transformation protocols (Cao *et al*., 2022, 2024; Fan *et al*., 2020; Lu *et al*., 2024; Zhang *et al*., 2023a). Although hairy roots induced by K599 exhibited relatively slower growth due to the lack of auxin genes in the agropine‐type TR‐DNA region ( Valdes Franco *et al*., 2016; Xiang *et al*., 2016) compared to C58C1, K599 may offer advantages for research on the biosynthesis of bioactive compounds and root development, as it eliminates the influence of auxin on these process (Zhang *et al*., 2023b).

Besides the variation of PAM and nucleotide composition surrounding the target sites caused by the experimental material and reference genome mentioned above, other factors including GC content, off‐target effects, specific nucleotide preferences near the PAM, and secondary structure of the sgRNA also influenced the editing efficiency in *S. miltiorrhiza*. Among the 65 target sites with HOM efficiency below 10%, 9 target sites have homologous genes with sequence similarity exceeding 80%, which may compete for repair templates during genome editing. Multiple factors (two or more) were found to contribute to their low efficiency, while most results align with previous studies, some sequence preferences showed distinct patterns. For example, G at position 19, C at 16, and C at 21 were more frequently associated with low‐efficiency groups, whereas T at position 21 was linked to high‐efficiency groups. These findings may provide critical insights for sgRNA design in *S. miltiorrhiza* and potentially other plants (Figure 6). Ultimately, our results indicate that 58 low‐efficiency targets (89.23%) can be attributed to known factors. The remaining seven targets without identifiable causes may reflect the fact that current sgRNA design rules are primarily derived from animal studies, and genomic context differences might hinder the extrapolation of comprehensive guidelines for plants.

## Materials and methods

### Materials

The purple‐flowered variety (ZH Danshen) and bh2‐7 tissue culture seedlings were obtained from our laboratory. *A. rhizogenes* strains Ar1193, Ar.Qual, MSU440, C58C1, and K599 were purchased from Shanghai Weidi Biotechnology Co., Ltd. MS medium (Phytotech), kanamycin sulfate (Kan), timentin (Tim), and streptomycin were acquired from Beijing Solarbio Science & Technology Co., Ltd. A fluorescence stereo microscope (ZEISS, Discovery.V20, Germany) was used for imaging. Plasmids pW501 and pZKD672 were kindly provided by Professor Huawei Zhang's lab at Peking University.

### Induction of hairy roots in bh2‐7 by different *A. rhizogenes* strains

The plasmid pW501 (Pan *et al*., 2022b), containing the red fluorescent protein DsRed was transformed into *A. rhizogenes* Ar1193, Ar.Qual, MSU440, and K599. These transformed strains were cultured at 28 °C for 2–3 days. Single colonies were selected, verified by PCR, and further cultured in a shaking incubator until the OD600 reached 0.6. The bacterial solution was centrifuged at 3000 ***g*** for 5 min and resuspended in MS medium for subsequent infection. Young leaves of bh2‐7 were excised into 1–2 cm^2^ pieces, infected with the prepared bacterial solution for 5 min, and then blotted dry on sterile filter paper to remove excess bacteria. The infected leaf explants were placed on MS medium and co‐cultivated under dark conditions at 25 °C for 2 days. Subsequently, the explants were transferred to a selective medium containing Kan and Tim to induce hairy root formation. Hairy roots typically emerged from the wound sites after approximately 2 weeks.

### Calculation of induction and positive rates of hairy roots in bh2‐7 by different *A. rhizogenes* strains

One month post‐infection, we assessed the induction rate (percentage of explants producing hairy roots), positive transformation rate (percentage of hairy roots expressing DsRed fluorescence), and the length of hairy roots induced by the different *A. rhizogenes* strains. Handheld fluorescence and confocal microscopy were used to visualize DsRed expression at 558 nm, with fluorescent explants considered successfully transformed. The positive induction rate was calculated using a minimum of 50 hairy roots from three explants for each strain. Additionally, the lengths of positive hairy roots from three explants per *A. rhizogenes* strain were measured using ImageJ to assess potential differences in growth patterns among the strains. All figures were generated using Excel and ImageGP (Chen *et al*., 2022).

### Target selection

A total of 241 sgRNAs targeting 131 genes were designed based on the bh2‐7 genome sequence, following previously established methods (Ma *et al*., 2015). Briefly, target sequences were selected to be 20 bp in length and located immediately upstream of the NGG PAM site, with a GC content between 50%–70%, using online software (http://skl.scau.edu.cn/home). Potential sgRNAs were further evaluated for predicted RNA stability and potential off‐target effects within the bh2‐7 genome. To confirm target specificity, genomic DNA was extracted from three sterile seedlings of the purple‐flowered variety and bh2‐7 variety. PCR amplification of the 241 target sites was performed using primers designed to flank each target site, followed by Sanger sequencing (Table S1).

### Construction of CRISPR/Cas9 knockout vector

The vector pZKD672 (Pan *et al*., 2022a) was digested with *BsaI*, and the resulting 15kb fragments was gel‐purified. Upstream and downstream target‐specific primers were annealed (95 °C for 30 s, 50 °C for 30 s, then held at 4 °C). The annealed primers and purified vector fragments were ligated overnight at 16 °C in a 10 μL reaction containing 1 μL 10 × T4 DNA ligase Buffer, 2 μL *BsaI* digested linearized vector, 5 μL annealed target fragment, 0.5 μL T4 DNA Ligase, and 2 μL ddH_2_O. The ligation products were transformed into *E. coli* DH5α competent cells and colonies were selected on LB agar plates containing kanamycin. Colony PCR using target‐specific primers and R50 primer (R50 primer sequence: 5′‐tgtcgaacaggagagcgcca‐3′) was used to identify clones containing the desired inserts. Positive clones were selected for plasmid extraction and sequencing to confirm the correct insertion of target sequences.

### Editing efficiency statistics for 53 targets in bh2‐7 by different *A. rhizogenes* strains

Plasmid contain the *SMILTO09119* target sequence was transformed into five *A. rhizogenes* strains (Ar1193, Ar.Qual, MSU440, C58C1, and K599), and hairy roots were induced as described previously. Additionally, 53 plasmids with different target sequences were transformed into C58C1 and K599. Genomic DNA was extracted from 1‐month‐old hairy roots, and PCR amplification of the target sites was performed using bh2‐7 specific primers (Table S1). PCR products were sequenced, and SnapGene software was used to analyse the sequences for mutations at the target sites. The online tool Synthego ICE (Inference of CRISPR Edits) was used for peak decomposition and mutant genotype analysis, providing genotype and indel scores for each mutant (Conant *et al*., 2022; Develtere *et al*., 2024; Enzmann and Wronski, 2018). Six parameters were evaluated for the five *A. rhizogenes* strains, including the average ICE score, editing efficiency, knockout (KO) efficiency, heterozygous mutation efficiency, chimera mutation efficiency, and homozygous or biallels (HOM) mutation efficiency.

## Author contributions

Mei Tian: Data curation, formal analysis, writing‐original draft and validation. Linglong Luo: Data curation and writing – review. Baolong Jin: Funding acquisition and writing review. Jianing Liu: Writing – editing and validation. Tong Chen: Data curation and formal analysis; Jinfu Tang: Software and visualization. Ye Shen: Materials and Software. Haiyan Zhang: Manuscript revised. Juan Guo: Data curation and Formal analysis. Huawei Zhang: Methodology, Software and writing – review. Guanghong Cui: Funding acquisition, project administration, conceptualization and resources. Luqi Huang: Funding acquisition, project administration, conceptualization and resources.

## Conflict of interest

The authors claim that the researchers in this study have no conflicts of interest.

## Supporting information


**Figure S1** Distinct mutation types were generated in five *A. rhizogenes* strains C58C1 (a), K599 (b), MSU440 (c), Ar.Qual (d), Ar1193 (e) using the *SMILTO09119* gene and pZKD672 vector system.

**Figure S2** Efficiency of targets with varying GC contents among 65 low‐efficiency targets.

**Figure S3** Secondary structures of canonical (a) and low efficiency sgRNA (b).

**Figure S4** Mutation profiles induced by C58C1‐mediated CRISPR/Cas9 targeting 150 sgRNAs. a. Mutation types and frequecy of biallelic or homozygous mutants across 150 sgRNAs. b. Nucleotide preference analysis for one base insertion.

**Table S1** Primers designed to flank the target sites with 241 targets.

**Table S2** Variation of 241 guide sequences across different materials and genomics.

**Table S3** Editing efficiencies of different mutation types in five *A. rhizogenes* strains using *SMILTO09119* gene and pZKD672 vector.

**Table S4** Editing efficiency for 20 targets in K599 and 33 targets in C58C1.

**Table S5** Editing efficiency for subsequent 117 targets in C58C1.

**Table S6** Factors affecting CRISPR/Cas9 editing efficiency in *S. miltiorrhiza*.

**Table S7** Mutation patterns 150 sgRNAs induced by C58C1.

## Data Availability

All the data supporting the findings of this study are available in the paper and supplementary data.
