# Supplementary material for: Highly efficient Agrobacterium rhizogenes‐mediated gene editing system in Salvia miltiorrhiza inbred line bh2‐7
Source: Plant Biotechnol J. 2025 Mar 26;23(6):2406–17. doi: 10.1111/pbi.70029 (PMC12120871; doi:10.1111/pbi.70029)
Supplement: Supplementary file 1 — Figure S1 Distinct mutation types were generated in five A. rhizogenes strains C58C1 (a), K599 (b), MSU440 (c), Ar.Qual (d), Ar1193 (e) using the SMILTO09119 gene and pZKD672 vector system. [file PBI-23-2406-s011.docx]

a b

C58C1


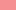
 **d<9
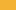
** **d>9
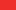
** **i**

K599

d<9
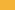
 d>9
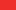
 i

30

**25.00**

**25.00**

**25.00**

**8.33**

**8.33 8.33**

25

Frequency(%)

20

15

10

5

0

d1 d3 d4 d5 d6 d>9

**25**

20

**19.57**

**17.39**

**10.87**

**15.22**

**10.87**

**8.70**

**4.35**

**4.35**

**2.17 2.17**

**2.17 2.17**

Frequency(%)

15

10

5

0

d1 d2 d3 d4 d5 d6 d7 d8 d>9 i1 i8 i>9

c MSU440 d

Ar.Qual


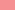
 **d<9
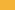
** **d>9
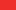
** **i
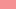
** **d<9
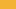
** **d>9
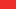
** **i**

30 40

**28.95**

**23.68**

**23.68**

**2.63 5.26**

**2.63 5.26 2.63 2.63 2.63**

**41.94**

**19.35**

**3.23 3.23**

**3.23**

**6.45 9.68 9.68**

**3.23**

Frequency(%)

Frequency(%)

30

20

20

10

10

0

d1 d2 d3 d4 d5 d6 d7 d9 d>9 i2

**0**

d1 d2 d3 d4 d5 d6 d7 d>9 i1

e Ar1193


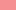
 **d<9
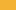
** **d>9
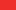
** **i**

30

**33.33**

**22.22**

**22.22**

**11.11**

**11.11**

Frequency(%)

20

10

0

d1 d2 d4 d>9 i1
