# Supplementary figures and images for: Highly efficient Agrobacterium rhizogenes‐mediated gene editing system in Salvia miltiorrhiza inbred line bh2‐7
Source: Plant Biotechnol J. 2025 Mar 26;23(6):2406–17. doi: 10.1111/pbi.70029 (PMC12120871; doi:10.1111/pbi.70029)

50


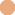

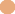

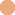

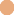

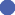

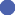

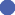

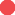

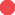

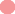

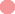

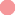

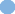

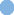

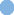

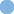


Efficiency(%)

#
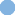
 GC-70

40
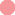
 GC-65

#
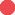
 GC-60

30
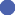
 GC-55

#
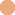
 GC-50

20

10

0

Supplement: Supplementary file 2 — Figure S2 Efficiency of targets with varying GC contents among 65 low‐efficiency targets. [file PBI-23-2406-s004.docx]
