# Supplementary material for: Highly efficient Agrobacterium rhizogenes‐mediated gene editing system in Salvia miltiorrhiza inbred line bh2‐7
Source: Plant Biotechnol J. 2025 Mar 26;23(6):2406–17. doi: 10.1111/pbi.70029 (PMC12120871; doi:10.1111/pbi.70029)
Supplement: Supplementary file 4 — Figure S4 Mutation profiles induced by C58C1‐mediated CRISPR/Cas9 targeting 150 sgRNAs. a. Mutation types and frequecy of biallelic or homozygous mutants across 150 sgRNAs. b. Nucleotide preference analysis for one base insertion. [file PBI-23-2406-s002.docx]

a b

**40**

30

**Insertion frequency(%)**

**30**

Mutant frequency(%)

20

**20**

**10 10**

0

d1 d2 d3 d4 d5 d6 d7 d8 d9 d>9 i1 i2 i>10

**0**

A T C G
